# Dissimilar Regulation of Antimicrobial Proteins in the Midgut of *Spodoptera exigua* Larvae Challenged with *Bacillus thuringiensis* Toxins or Baculovirus

**DOI:** 10.1371/journal.pone.0125991

**Published:** 2015-05-18

**Authors:** Cristina M. Crava, Agata K. Jakubowska, Baltasar Escriche, Salvador Herrero, Yolanda Bel

**Affiliations:** 1 Department of Genetics, University of Valencia, Burjassot, Valencia, Spain; 2 Estructura de Recerca Interdisciplinar en Biotecnologia i Biomedicina (ERI BIOTECMED), University of Valencia, Burjassot, Valencia, Spain; University of Tennessee, UNITED STATES

## Abstract

Antimicrobial peptides (AMPs) and lysozymes are the main effectors of the insect immune system, and they are involved in both local and systemic responses. Among local responses, midgut immune reaction plays an important role in fighting pathogens that reach the insect body through the oral route, as do many microorganisms used in pest control. Under this point of view, understanding how insects defend themselves locally during the first phases of infections caused by food-borne pathogens is important to further improve microbial control strategies. In the present study, we analyzed the transcriptional response of AMPs and lysozymes in the midgut of *Spodoptera exigua* (Lepidoptera: Noctuidae), a polyphagous pest that is commonly controlled by products based on *Bacillus thuringiensis* (Bt) or baculovirus. First, we comprehensively characterized the transcripts encoding AMPs and lysozymes expressed in *S*. *exigua* larval midgut, identifying 35 transcripts that represent the *S*. *exigua* arsenal against microbial infection. Secondly, we analyzed their expression in the midgut after ingestion of sub-lethal doses of two different pore-forming *B*. *thuringiensis* toxins, Cry1Ca and Vip3Aa, and the *S*. *exigua* nucleopolyhedrovirus (SeMNPV). We observed that both Bt toxins triggered a similar, wide and in some cases high transcriptional activation of genes encoding AMPs and lysozymes, which was not reflected in the activation of the classical systemic immune-marker phenoloxidase in hemolymph. Baculovirus ingestion resulted in the opposed reaction: Almost all transcripts coding for AMPs and lysozymes were down-regulated or not induced 96 hours post infection. Our results shed light on midgut response to different virulence factors or pathogens used nowadays as microbial control agents and point out the importance of the midgut immune response contribution to the larval immunity.

## Introduction

Antimicrobial peptides (AMPs) and lysozymes are small proteins produced by a wide range of organisms (from bacteria to humans) which have direct antimicrobial activity against pathogens like bacteria, fungi and viruses [1,2]. In eukaryotes, they are part of the innate immune system and they act as chemical effectors to arrest microbial infections. In insects, they are induced after pathogen recognition and they are involved in two different innate immune defenses: the local and humoral responses [3]. Insects rely on both immune responses to fight infections, and one of the main differences between them is that their effects are either local or systemic.

Local defenses are constituted by physical barriers that prevent microbial penetrance (such as epithelia), and by local production of AMPs, lysozymes and reactive oxygen species, at the site of infection. Hence, AMPs and lysozymes represent one of the first chemical barriers against infections. Systemic defenses are activated later and include a series of orchestrated mechanisms which range from the production of AMPs and lysozymes by the fat body, which are finally secreted into hemolymph to clear invading microbes inside body cavity (humoral response), to the cellular response lead by specialized cells like hemocytes (phagocytosis and encapsulation of foreign intruders), to the melanization and coagulation of the hemolymph [3,4]. Since the discovery of the first insect AMP [5], the role of antimicrobial proteins in humoral response has been broadly investigated [2], however their contribution to the local response was discovered later [6] and to date it has only been studied in model organisms [3,7,8]. Besides their functions in the direct clearance of invading microbes in immune response, antimicrobial proteins like AMPs and lysozymes also exert other important functions as immuno-modulatory activities [9] and maintenance of the endosymbiotic bacteria homeostasis [10].

Since the first AMP was discovered in the 1980s in a giant silk moth [5], a large number of AMPs have been identified to date. Insect AMPs generally share common features: they are cationic molecules of less than 100 amino acid residues, and their expression is generally induced by injury or microorganism infection [2]. The importance of AMPs and lysozymes in insects defense is thought to be reflected in the expansion of genes coding for such proteins in insect genomes [11]. Accordingly to this hypothesis, the increasing availability of transcriptomic and genomic data from several insect species has led to the discovery of a variety of immune-related genes which highlights the evolutionary diversity and amplitude of the AMP arsenal in insects. It has been proposed that expansion or loss of AMPs in different insect species is a consequence of the different pathospheres in which insects live, and that the remarkable plasticity of genes coding for AMPs represents an adaptive response to biotic and abiotic stresses [12]. To date, the best characterized AMPs are those from the model organism *Drosophila melanogaster*, whose response patterns are well known. About 20 immune-inducible AMPs, which can be grouped in seven classes, are expressed in *D*. *melanogaster* after immune-challenges. Diptericins, drosocins and attacins are very effective against Gram-negative bacteria. Defensins are important for killing Gram-positive bacteria whereas drosomycins and metchnikowins are induced after fungal infection. Finally, cecropins have both antibacterial and antifungal properties [3,4]. In another model organism, the Lepidoptera *Bombyx mori*, around 30 AMPs and four lysozymes have been described so far [11].

*Spodoptera exigua* (Hübner) is a polyphagous pest that attacks many important economic crops, such as cotton, alfalfa, tomato, sugar beet and many ornamental plants worldwide. It has been controlled by chemical pesticides during decades leading to the problem of development of resistance [13]. This, together with the public concerns about the ecological risks related with the use of chemical pesticides, has promoted the adoption of more eco-friendly methods. Nowadays, two effective microbial alternatives are commercially available for the control of *S*. *exigua*: products containing *Bacillus thuringiensis* (Bt) Berliner spores and crystal toxins or baculovirus-based insecticides. *B*. *thuringiensis* is a Gram-positive bacterial pathogen that produces several insecticidal toxins during its life cycle, which target the midgut epithelium of susceptible insects (mainly larval stages from orders Coleoptera, Lepidoptera and Diptera). The most studied, as well as the most used Bt toxins, are called Cry proteins, which are produced as crystal inclusions during sporulation [14]. Cry toxins are widely used in countries that allow transgenic crop cultivation, since they are expressed in transgenic plants to provide resistance against several Lepidoptera and Coleoptera pests. A second class of Bt toxins (also expressed in transgenic plants) includes Vip proteins, which are soluble proteins produced during the vegetative phase of *B*. *thuringiensis* life cycle [15]. Toxins from both classes are toxic after ingestion and although their mode of action has not been completely clarified, is thought that they both act as pore-forming toxins, inserting in the midgut epithelial membrane of susceptible insects and causing an osmotic shock that leads to cellular death. Whereas the mode of action of Cry proteins has been extensively studied and several Cry-binding proteins have been identified from the midgut epithelia [16], little is known about the details of the mode of action of Vip proteins [17,18]. Baculoviruses are large DNA viruses that infect arthropods, mainly from the orders Lepidoptera, Diptera and Hymenoptera. During their replication cycle, they produce two distinct morphological and functional forms, the occlusion-derived viruses (ODVs) and the budded viruses [19]. Several ODVs are normally included in a single polyhedron occlusion body (OB) which is orally consumed by larvae in natural conditions. The alkaline environment of larval midgut favors ODV release, which subsequently cross the peritrophic membrane and fuse with the cell membrane of midgut epithelial cells. The virus replicates in these cells forming budded viruses that allow for systemic spread of the infection within the host [20].

Larval midgut is targeted by both *B*. *thuringiensis* and baculovirus, which use it as portal of entry to later establish a systemic infection within insect body; hence midgut immune response may play an important role in defense against both pathogens, and contribute to the success or failure of the infection at early phases. A better knowledge of the diversity of antimicrobial proteins expressed in the larval midgut as well as the deciphering of their specific activity in midgut immune response during food-borne pathogen challenge will broaden our understanding of how caterpillars have adapted to pathogens and environment. Studies which include insects other than model organisms, especially crop pest species which are routinely controlled by microbial pesticides, will shed light on the insect response to the microbes currently used in insect control, and potentially lead to the improvement of the microbial insecticides.

In the present study we focused on the characterization of the main arsenal of antimicrobial proteins (AMPs and lysozymes) expressed in the midgut of *S*. *exigua*. We first characterized transcripts coding for AMPs and lysozymes, describing for the first time several new AMPs expressed in this insect. Quantitative real-time RT-PCR (qRT-PCR) was then used to determine the local regulation of a large subset of AMP and lysozyme transcripts in larvae after sub-lethal oral intoxication with diverse Bt virulence factors like Cry1Ca and Vip3Aa toxins, and with a specialized pathogen, the *S*. *exigua* baculovirus (SeMNPV). Our results showed that midgut transcriptional response of antimicrobial proteins was wide and robust when Bt pore-forming toxins were ingested in contrast to what happened when the ingested pathogen was a baculovirus, which exerts toxicity through a completely different process.

## Materials and Methods

### Sequence mining and phylogenetic analyses

*S*. *exigua* transcriptome [21] was mined for the presence of unigenes coding for AMPs and lysozymes. Similar nucleotide sequences were aligned with ClustalX 2.1 with default settings [22] and visualized in GeneDoc [23]. If necessary, alignments were manually edited (in case of misleading assemblies which resulted in unigenes containing two coding sequences with two different Blast hits or by removing zones with Phred values lower than 20). In order to obtain a non-redundant set of transcripts, unigenes with nucleotide similarity higher than 90% (comprising both ORF and UTRs) were considered as alleles of the same gene. The existence of the assembled unigenes coding for AMPs and lysozymes was confirmed by looking for their presence in our Sanger library [21] (S1 Table). In case that no correspondent sequences were present in the Sanger library, we designed specific primers to amplify *S*. *exigua* cDNA and confirm the presence of such AMP or lysozyme by sequencing (S2 Table). Hence, all the sequences described in this study were verified by Sanger sequencing therefore discarding the possibility that artifacts derived from misleading contig assembly could be present. However, it should be noted that we cannot discard that some transcripts might be allelic variants of the same gene, since heterogeneity in immune system genes is highly correlated with pathogen diversity via host-parasite coevolution [24]. Different pathogens can maintain host genetic variation by favoring the distribution of alternative genotypes [12] and these could be reflected in our transcriptome containing RNA from three different *S*. *exigua* colonies [21].

Amino acid sequences, as well as molecular weight of the putative proteins, were predicted by the EditSeq software (DNAStar, Madison, WI, US). Predicted amino acid sequences were aligned with MUSCLE software [25] and visualized in GeneDoc. Phylogenetic analyses were carried out using the neighbor-joining algorithm with p-distance method and 100 bootstrap replicates on the MEGA5 program [26]. Phylogenetic trees were visualized with MEGA5. Sequences from other species employed in phylogenetic analyses were retrieved after Blast-search against different databases: NCBI’s non-redundant database (nr), SPODOBASE (http://bioweb.ensam.inra.fr/spodobase/) and Silkworm Genome Database (http://silkworm.genomics.org.cn/). To obtain *Spodoptera frugiperda* amino acid sequences, we used *S*. *exigua* nucleotide sequences as queries in the Blast search implemented in SPODOBASE. For each search, the ORF from the sequence with the highest e-value was translated to protein using the software EditSeq and used for the analysis. All the *S*. *exigua* sequences identified in this study have been deposited in GenBank (accession numbers KP056508-KP056542).

### Insects

*S*. *exigua* larvae challenged with Bt toxins were obtained from the FRA colony, which was kindly provided by Dr. M. López-Ferber (INRA, St.-Christol les Alés, France) [27] and maintained at the University of Valencia (Spain). Baculovirus-free *S*. *exigua* larvae were kindly provided by Dr. P. Caballero from the Public University of Navarra (Pamplona, Spain). The Baculovirus-free colony was regularly checked for the presence of baculovirus infection by qRT-PCR and proved to be negative for SeMNPV. Both insect colonies were reared at 25°C, relative humidity of 70% and a photoperiod of 16 h:8 h (light:dark), on artificial diet, as described in Bel *et al*. [28].

### Detection of AMP and lysozyme transcripts in larval midgut

To ensure that all the transcripts identified after transcriptome sequence mining were expressed in *S*. *exigua* larval midgut, specific primers were designed (S2 Table). cDNA was prepared from total RNA extracted from larval midguts using RNAzol RT reagent (Sigma-Aldrich, St. Louis, MO, US), following the manufacturer’s instructions. Purified RNA was then treated with DNase I (Invitrogen, Carlsbad, CA, US) and subsequently reverse-transcribed to cDNA using oligo-(dT) primers and the SuperScript II Reverse Transcriptase (Invitrogen), according to the manufacturer's protocol. PCRs were carried out in a final volume of 25 μl containing 400 ng of cDNA, 0.75 U of Taq polymerase (Biotools, Madrid, Spain), 1X reaction buffer, 160 μM dNTPs and 0.32 μM of each primer. Amplification conditions were the following: 94°C for 5 min, followed by 35 cycles of 94°C for 30 s, 60°C for 1 min and 72°C for 1 min and a final elongation at 72°C for 5 min. Correct amplicon sizes were checked after running PCR products in a 2% agarose gel stained with GelRed (Biotium, Hayward, CA, US).

### Bt toxin production and purification

Vip3Aa protein was produced by an *Escherichia coli* WK6 strain which harbored the recombinant plasmid pGA85, kindly supplied by Bayer CropScience N.V. (Ghent, Belgium). Protein production, lysate preparation and Vip3Aa quantification were carried out as described by Bel *et al*. [28]. Filtered lysates were stored at -20°C until use. Cry1Ca protein was also produced by an *E*. *coli* WK6 strain, which contained the recombinant plasmid pDB150, kindly supplied by Dr. R. de Maagd (Wageningen University, the Netherlands). Inclusion body purification, Cry1Ca protein solubilization and trypsin activation were performed according to the protocol described by Herrero *et al*. [29]. Cry1Ca protein was subsequently purified by anion-exchange chromatography using the ÅKTA Explorer System (GE Healthcare Life Sciences, Uppsala, Sweden). The protein was first dialyzed overnight at 4°C against 20 mM Tris/HCl buffer (pH 8.6), centrifuged and filtered before to be loaded on a 5 ml HiTrap Q column (GE Healthcare Life Sciences). Cry1Ca was then eluted from the column by a NaCl gradient. Fractions were recovered and checked for the presence of Cry1Ca by 12% SDS-PAGE. Fractions containing Cry1Ca were pooled, quantified by Bradford assay and stored at -20°C until use.

### Bt toxin treatments

Treatments with Bt toxin were carried out according the method previously used to analyze the response of *S*. *exigua* to Vip3Aa toxin by microarray analysis [28]. We used newly molted L4 larvae (±16 h) and toxin concentrations able to produce 99% growth inhibition. In the case of Vip3Aa we used a concentration of 111 ng/cm^2^ [28] whereas in the case of Cry1Ca we used 900 ng/cm^2^ (S1 Fig). To obtain results comparable to those described by Bel *et al*. (2013) [28], we used crude *E*. *coli* lysate containing Vip3Aa. However, Cry1Ca protein has different biochemical properties than Vip3Aa and it is produced as an insoluble protein (inclusion body) in *E*. *coli*. Hence, to achieve results comparable between the two toxins, Cry1Ca protein previously purified by anion-exchange chromatography was mixed with a known concentration of crude lysate total proteins from an empty *E*. *coli* WK6 strains (S2 Fig). The crude lysate from the empty *E*. *coli* WK6 was then used as control for both Vip3Aa and Cry1Ca, as well as the phosphate buffer (pH 7.4) containing 0.5 M NaCl used for cell lysis.

Three independent replicates were performed for each treatment. In each replicate, 16 weighed larvae were exposed to surface-contaminated diet placed in 2 cm^2^ wells of a 128-well bioassay tray (Bio-Cv-16, C-D International, Pitman, NJ, US). After 24 h larvae were weighted again to calculate growth inhibition using the formula previously described [30] and six larvae of each treatment were selected for hemolymph extraction and gut dissection. After dissection, guts were pooled and total RNA was purified using RNAzol RT reagent, following the manufacturer′s protocol. RNA was first treated with Recombinant DNase I from Takara (Saint-German-en-Laye, France) and then 2 μg of DNase-treated RNA were reverse-transcribed to cDNA using the PrimeScript II 1st strand cDNA Synthesis Kit (Takara), following the manufacturer′s instructions. To improve cDNA synthesis from total RNA, each reaction included both oligo-(dT) primers and random 6-mers.

### Baculovirus treatment

*S*. *exigua* newly molted L4 larvae were individually challenged with SeMNPV at a dose of 10^4^ OBs/larvae by adding virus suspension to a diet plug, as previously described [31]. Control larvae were fed with water. Five larvae were used per each treatment and condition, and the experiment was performed in triplicate. Guts from infected and control larvae were collected at 96 hours post infection (hpi), pooled, and used for total RNA extraction using RNAzol RT reagent. 1 μg of RNA was used for cDNA synthesis. RNA was first treated with DNase I (Invitrogen) and subsequently reverse-transcribed to cDNA using oligo-(dT) primer and SuperScript II Reverse Transcriptase (Invitrogen) according to the manufacturer's protocol.

### Quantitative real-time RT-PCR (qRT-PCR)

Primers for gene expression analysis were designed using Primer Express Software from Applied Biosystems (Carlsbad, CA, US) (S2 Table) and synthesized by Sigma-Aldrich. For families whose members showed a high degree of similarity (e.g. cecropins or diapausins), regions with high variability were used (most of them corresponding to the 3’end of the ORFs and to the first part of the 3’UTRs). However, in the case of c-LYZ1 the amplified fragment comprised the three isoforms 1A, 1B and 1C. Similarly, the amplified fragment for cecropin F covered both isoforms F1 and F2. Subunit C of ATP synthase was used as reference gene for normalization [28,31,32]. qRT-PCR reactions were carried out in optical 96-well plates (Applied Biosystems) on an ABI PRISM 7000 machine (Applied Biosystems) using the EvaGreen dye (Biotis, Vilnius, Lithuania), following the manufacturer's instructions. Each reaction was performed in a total volume of 20 μl, which contained 4 μl of cDNA diluted 1:10 (corresponding to ~ 300 ng). Forward and reverse primers were added to a final concentration of 300 pM. The specific amplification of transcripts was verified by dissociation curve analysis. Each PCR reaction was run twice if the SD between the two replicates was lower than 0.35 and if the Cts were not higher than 30, otherwise further replicates were added. Prior to quantifying differential expression among different treatments, the efficiency of each pair of primers was evaluated by performing 5-fold dilution series experiments (S3 Table). Primer efficiencies were taken into account during calculation of the expression ratios (-fold change) by using the REST MCS software (version 2) [33]. Only in the case of c-LYZ3, we used a default efficiency value of 2 since it was not possible to test primer efficiency because the transcript was always expressed at very low levels in all tested tissues. Results were visualized with GraphPad Prism v. 5.1 (GraphPad Inc., La Jolla, CA, US).

### Phenoloxidase assays

Hemolymph phenoloxidase (PO) activity was determined using 2 μl of hemolymph obtained from each individual larva as previously described [34]. Hemolymph was collected, immediately diluted 1:10 in ice-cold PBS (137 mM NaCl, 2.7 mM KCl, 10 mM Na_2_HPO_4_, 1.8 mM KH_2_PO_4_, pH7.4) and centrifuged at 16,000 *x g* for 3 min at 4°C to obtain cell-free plasma. Samples were then snap frozen in liquid N_2_ and conserved at -80°C until use. PO activity was assayed by adding 8 μl of cell-free plasma to 200 μL of 1 mM L-Dopa (Sigma-Aldrich) in PBS. Measurements were taken by an ELISA Multiskan Ascent reader (Thermolab Systems) at 490 nm every minute throughout 45 minutes. Since the observed absorbance curve was linear from 20–45 min after adding the substrate, the slope of the curve from 25–45 min of the reaction was used to calculate the absorbance increase per min. Measurements were repeated twice for each sample.

## Results and Discussion

### Identification of *S*. *exigua* transcripts encoding AMPs and lysozymes in larval midgut

Extensive data mining of the *S*. *exigua* transcriptome [21] was performed to identify transcripts encoding AMPs and lysozymes. The effort resulted in the identification of a wide and diverse spectrum of antimicrobial proteins that constitute a considerable part of the defensive arsenal of *S*. *exigua* against pathogen infection (Table 1).

**Table 1:** List of the different classes of transcripts coding for antimicrobial proteins retrieved from *S. exigua* transcriptome [21] and expressed in larval midgut.

Type of transcript	n of unigenes in *S*. *exigua* transcriptome (with complete ORF)	ORF size (bp)	aa mature protein/pro-protein	Molecular Mass (kDa)	Best Blastp hit^a^	E-value
**Glycine-rich AMPs**						
*Se*Gloverin	10 (3)	528	157	17.4	*S*. *exigua* ADL27731	3e-109
*Se*Attacin1	23 (3)	765	237	25.9	*Trichoplusia ni* P50725	1e-105
*Se*Attacin2	4 (4)	708	219	24.2	*Heliothis virescens* ACR78454	5e-97
*Se*Attacin3	6 (2)	708	219	24.2	*H*. *virescens* ACR78454	3e-82
**Cysteine-rich AMPs**						
*Se*Defensin (*Se*Spodoptericin)	8 (1)	309	78	8.5	*S*. *exigua* AEW24427	5e-50
*Se*Gallerimycin	5 (2)	228	55	6.2	*S*. *exigua* ADJ95798	1e-31
*Se*X-tox	6 (0)^b^	1470^b^	472	52.7	*S*. *frugiperda* AFC87713	0.00
*Se*Cobatoxin A	12 (4)	261	69	7.7	*S*. *frugiperda* AAQ18900	3e-46
*Se*Cobatoxin B	4 (3)	375	108	11.6	*T*. *ni* ABV68874	2e-14
*Se*Diapausin A1	4 (4)	192	40	4.5	*Spodoptera litura* ABU96713	3e-21
*Se*Diapausin A2	2 (2)	192	40	4.5	*S*. *litura* ABU96713	6e-22
*Se*Diapausin A3	2 (2)	192	40	4.5	*S*. *litura* ABU96713	6e-22
*Se*Diapausin A4	2 (2)	192	40	4.5	*S*. *litura* ABU96713	6e-22
*Se*Diapausin A5	2 (1)	192	40	4.5	*S*. *litura* ABU96713	6e-22
*Se*Diapausin A6	2 (2)	192	40	4.5	*S*. *litura* ABU96713	6e-22
*Se*Diapausin A7	2 (1)	192	40	4.5	*S*. *litura* ABU96713	6e-22
*Se*Diapausin B1	2 (1)	192	40	4.5	*S*. *litura* ABU96713	1e-17
**Proline-rich AMPs**						
*Se*Lebocin1	4 (1)	474	136	15.6	*Galleria mellonella* ACQ99193	5e-46
*Se*Lebocin2	2 (1)	477	137	15.4	*G*. *mellonella* ACQ99193	6e-36
**Amphipathic peptides**						
*Se*Cecropin A1	10 (4)	192	41	4.5	*S*. *litura* ABQ51092	1e-19
*Se*Cecropin A2	4 (2)	192	41	4.5	*S*. *litura* ABQ51092	1e-19
*Se*Cecropin B1	3 (3)	189	40	4.3	*S*. *litura* Q9XZG9	8e-19
*Se*Cecropin C	8 (5)	192	42	4.5	*Helicoverpa armigera* AAX51193	2e-13
*Se*Cecropin D	3 (2)	192	41	4.5	*S*. *litura* Q9XZH0	8e-20
*Se*Cecropin E	1 (1)	192	41	4.3	*B*. *mori* NP*_*001037392	3e-13
*Se*Cecropin F1	4 (2)	189	40	4.3	*S*. *litura* Q9XZH0	1e-17
*Se*Cecropin F2	5 (2)	189	40	4.3	*S*. *litura* Q9XZH0	7e-18
*Se*Moricin	8 (3)	204	45	4.8	*S*. *exigua* AAT38873	3e-22
**Lysozymes**						
*Se*C-LYZ1A	11 (3)	426	121	14.0	*S*. *exigua* AAP03061	2e-85
*Se*C-LYZ1B	2 (1)	426	121	14.0	*S*. *exigua* AAP03061	7e-85
*Se*C-LYZ1C	2 (1)	426	121	14.0	*S*. *exigua* AAP03061	2e-85
*Se*C-LYZ2	3 (2)	426	121	13.9	*Pseudoplusia includens* AAS48094	3e-59
*Se*C-LYZ3	1(0)^c^	420^c^	120	13.6	*Ostrinia furnacalis* AGV28584	4e-49
*Se*LLP1	9 (2)	552	157	18.4	*Antheraea*. *mylitta* ABP52098	2e-87
*Se*LLP2	4(1)	579	171	20.0	*Danaus plexippus* EHJ75441	2e-50

^a^Blastp search performed on August 2014.

^b^No unigenes with homology to pre-pro-protein x-tox including a complete ORF were found. Complete ORF was retrieved by assembling the six unigenes with Seqman software (Lasergene, DNASTAR Inc.).

^c^C-LYZ3 cluster includes only one unigene that does not contain a complete ORF. Searches with Blastn tools retrieved a complementary sequence (GenBank acc. nr. GAFU01015304) that was further assembled with the c-LYZ3 sequence of the present work to obtain a consensus sequence containing a full-length ORF. The existence of the full length transcript was confirmed by PCR and sequencing.

All the transcripts identified after transcriptome sequence mining were amplified using cDNA synthesized from midgut RNA, revealing that these antimicrobial proteins are actively transcribed in *S*. *exigua* midgut in normal conditions. This may be due to the need for broad spectrum local response molecules to control different food-borne pathogens which may continuously reach the insect body cavity through midgut, since this is the only part of the gastrointestinal tract that is not covered by a cuticular layer. Moreover, feeding behavior of lepidopteran larvae exposes them to a wider range of potential pathogens compared to piercing-sucking insects, since they ingest a high quantity of plant material including plant surface, where a large number of bacteria is most probably located. Also, a pool of diverse antimicrobial proteins may be needed in midguts to maintain homeostasis of the commensal bacteria community since lepidopteran larvae seem to be able to shape the phylotypes of the gut microbiota, as recently reported [35]. In any case, our results show that *S*. *exigua* midgut expresses a large set of transcripts encoding antimicrobial proteins, highlighting the important role of these peptides in larval midgut homeostasis and immunity.

It has been recently proposed that, amongst insects, generalist herbivores may have a more efficient immune defense strategy than specialist herbivores [36], and in the case of *S*. *exigua* this may be reflected in the expansion of AMP and lysozyme gene families. Further analyses of the EST database SPODOBASE revealed that such an arsenal is widely shared with another generalist related species, *S*. *frugiperda*, suggesting a common evolution for AMPs and lysozymes between these two species. Below, the different families of *S*. *exigua* AMPs and lysozymes with the description of their main features are reported, including the new transcripts retrieved in this work.

#### Glycine-rich AMPs

**Fig 1:**
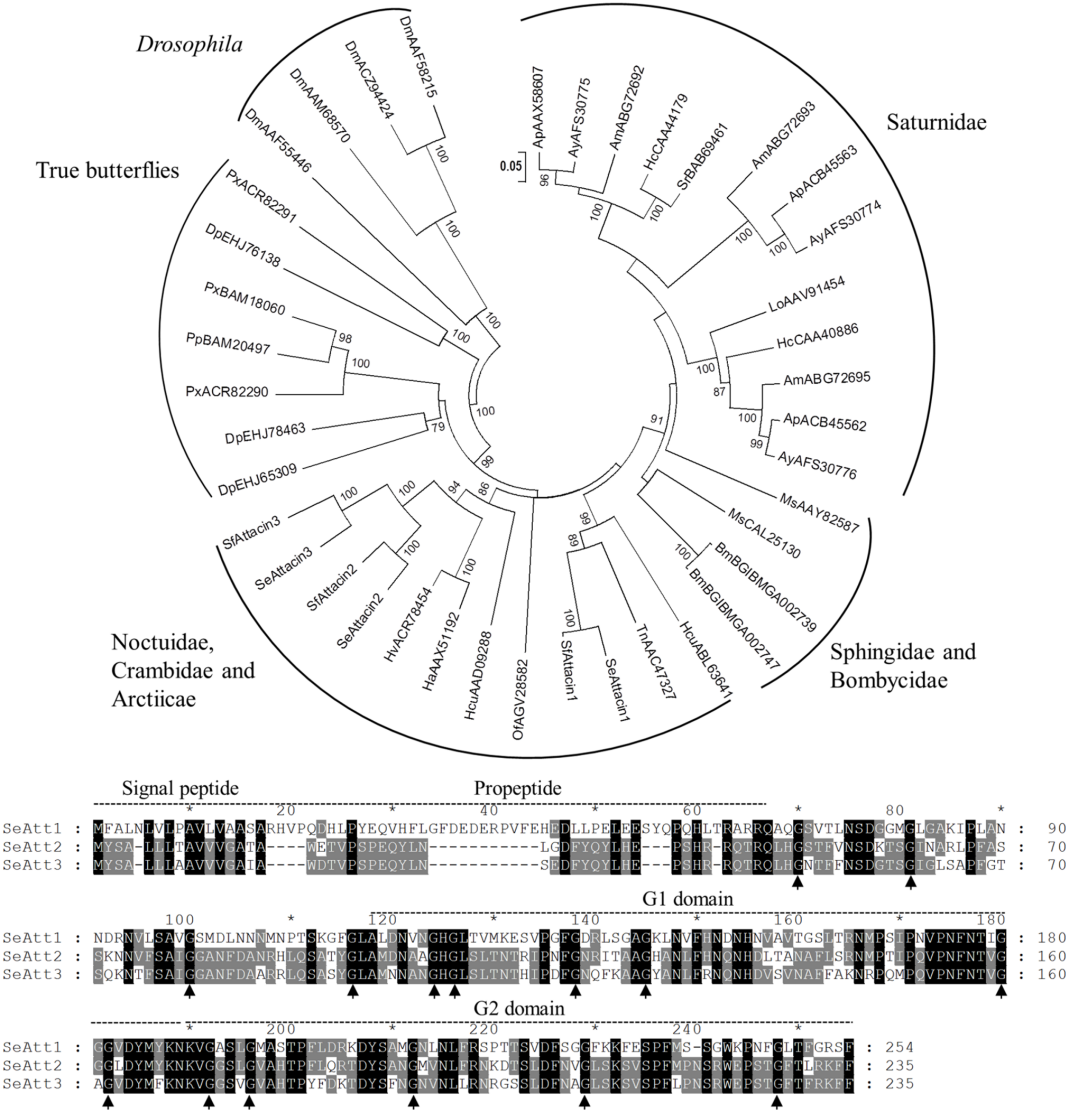
Phylogenetic analysis of attacin proteins from Lepidoptera. Upper panel) Neighbor-joining tree of Lepidoptera attacins. *D*. *melanogaster* attacins were used as outgroup. Bootstrap values (above 75) are indicated on each node of the tree (100 replicates). Sequences from nr database, SPODOBASE and Silkworm Genome Database were used. Accession number of each sequence is indicated. Abbreviations: Am: *A*. *mylitta*, Ap: *Antheraea pernyi*, Ay: *Antheraea yamamai*, Bm: *B*. *mori*, Dm: *D*. *melanogaster*; Dp: *D*. *plexippus*, Ha: *H*. *armigera*, Hv: *H*. *virescens*, Hc: *H*. *cecropia*, Hcu: *Hyphantria cunea*, Lo: *Lonomia obliqua*, Ms: *Manduca sexta*, Of: *O*. *furnacalis*, Pp: *Papilio polytes*, Px: *Papilio xuthus*, Sc: *Samia cynthia*, Se: *S*. *exigua*, Sf: *S*. *frugiperda*, Tn: *T*. *ni*. Lower panel) ClustalX alignment of *Se*Attacins. Black shadows highlight residues conserved between the three isoforms whereas grey shadows highlight residues conserved among two isoforms. Arrows indicate conserved glycine residues.

In Lepidoptera, two classes of glycine-rich AMPs have been identified: attacins and gloverins. It has been reported that both mainly target Gram-negative bacteria and fungi [2]. Attacins have been found in both Lepidopteran and Dipteran species whereas gloverins are typical of Lepidoptera. Attacins were first purified from the hemolymph of *Hyalophora cecropia* and separated in two groups based on their amino acid properties: the basic and the acidic isoforms [37]. Our transcriptome data mining led to the identification of three transcripts encoding attacins in *S*. *exigua* (Table 1), in contrast to the two genes present in *B*. *mori* genome [11]. *Se*Attacin1 (previously described by Bang *et al*., [38]) corresponded to the acidic form (calculated pI 6) whereas *Se*Attacin2 and *Se*Attacin3 represented the basic forms (calculated pI 9). Homologs from *S*. *frugiperda* were retrieved by Blast searches in SPODOBASE (acc. nr. *Sf*Attacin1, Sf2H08805-3-1; *Sf*Attacin 2, Sf1P24355-5-1; *Sf*Attacin 3, Sf2H07836-5-1) and amino acid identities between the two species ranged from 85 to 90%. Phylogenetic analysis revealed that lepidopteran attacins formed a highly divergent gene family, with no close orthologs or apparent subfamily structure conserved across lepidopteran families (Fig 1).

Gloverins are Lepidoptera glycine-rich AMPs that have not been identified in other insect orders [2]. Up to four different gloverin genes have been found in *B*. *mori* genome so far [11] whereas in the *S*. *exigua* transcriptome only one transcript coding for gloverin was identified (Table 1), which corresponded with the gene previously described by Hwang & Kim [39]. The *in silico* translated protein shared 92% identity with its *S*. *frugiperda* homolog (acc. nr. Sf2H01396-5-1), retrieved from SPODOBASE after Blast search.

#### Cysteine-rich AMPs

Cysteine-rich AMPs are cationic AMPs widely distributed among different organisms, from plants to humans. The presence of at least six cysteine residues forming at least three disulfide bridges is their main structural feature [40]. Within insect class, the best-studied cysteine-rich AMPs have six conserved cysteine residues which form a cysteine-stabilized alpha beta motif (CS-αβ). CS-αβ lepidopteran AMPs have been well studied in *S*. *frugiperda* and four different transcripts belonging to four different classes have been described [41,42]. In *S*. *exigua* transcriptome, we found up to five transcripts coding for CS-αβ AMPs that could be grouped into four classes. For each of the first two classes, *Se*Defensin (alternatively named *Se*Spodoptericin according to nomenclature in *Spodoptera* genus) and *Se*Gallerimycin, we identified only one transcript, as described in *S*. *frugiperda*. The amino acid identities between the predicted proteins and their *S*. *frugiperda* homologs (acc. nr. AAQ18895 and AAQ18896, respectively) were 93 and 85%, respectively. Interestingly, in *B*. *mori* genome only one gene coding for defensin has been retrieved, whereas no gallerimycin orthologs have been found so far [11].

Another class of CS-αβ lepidopteran AMPs are the x-tox proteins, which are large pre-pro-proteins composed by several CS-αβ motifs (the structural scaffold of invertebrate defensins) [42]. Only one transcript coding for *Se*X-tox was retrieved in *S*. *exigua* transcriptome, and it was slightly different from its ortholog from *S*. *frugiperda* (acc. nr. AFC87713). *S*. *frugiperda* x-tox is composed of 11 CS-αβ whereas the *in silico* translated sequence from *S*. *exigua* only had 8 repeats (S3 Fig). It is thought that genes encoding x-tox in Lepidoptera undergo accelerated evolution, by acquisition of new repeat motifs or by duplication of preexisting repeat motifs. To support this hypothesis, besides finding different numbers of CS-αβ motifs between species (six in *G*. *mellonella*, five to six in *B*. *mori* and three in *H*. *virescens*), it has been shown that the number of CS-αβ motifs in *S*. *frugiperda* can also vary between individuals from the same species [43]. Hence, although we identified an 8 repeat *Se*X-tox, we cannot rule out that this protein may be present with a different number of repeats in other *S*. *exigua* individuals or populations.

**Fig 2:**
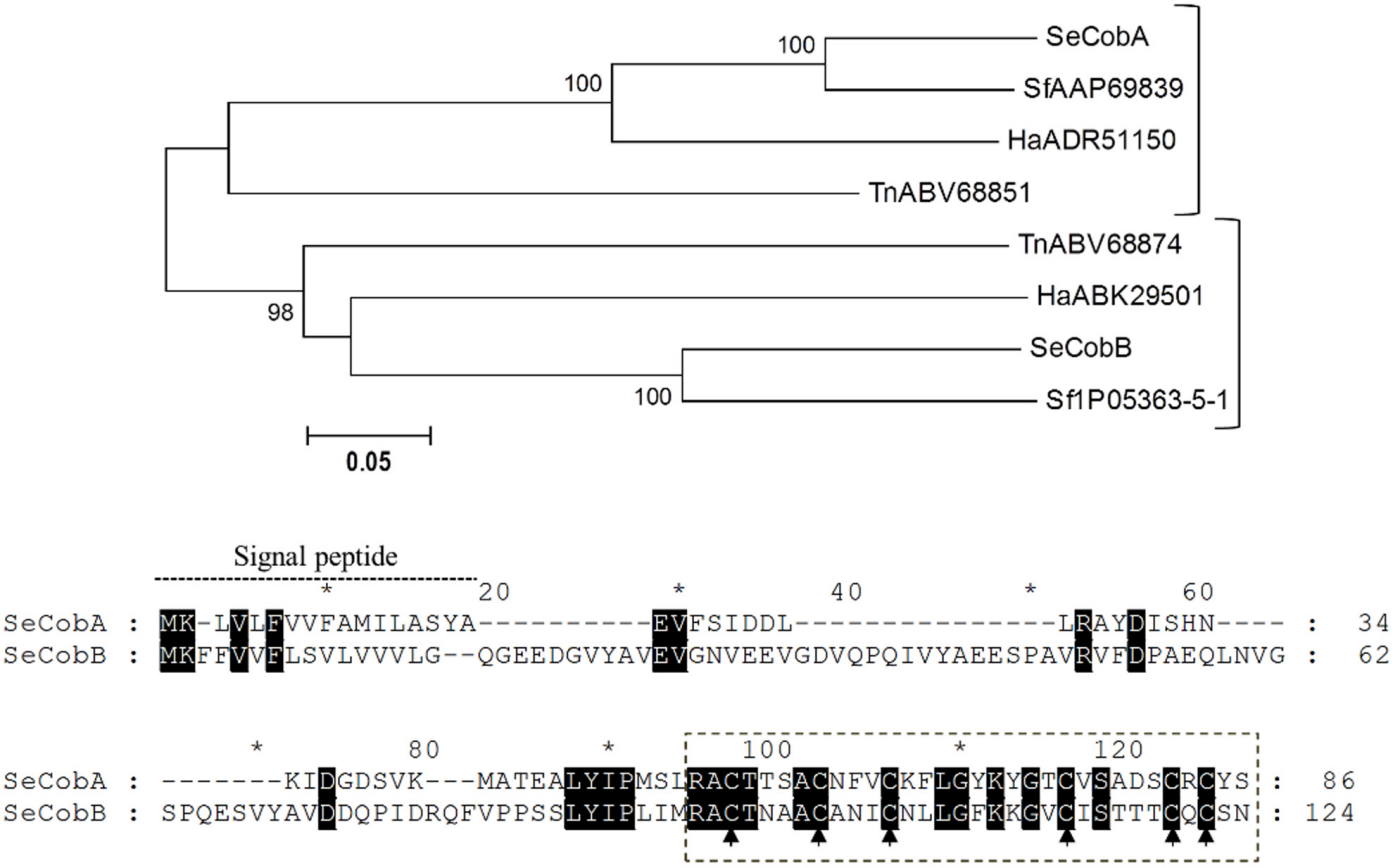
Phylogenetic analysis of cobatoxin proteins from Lepidoptera. Upper panel) Neighbor-joining tree of Lepidoptera cobatoxins. Bootstrap values (above 75) are indicated on each node of the tree (100 replicates). Sequences from nr database and SPODOBASE were used. Accession number of each sequence is indicated. Ha: *H*. *armigera*, Se: *S*. *exigua*, Sf: *S*. *frugiperda*, Tn: *T*. *ni*. Lower panel) ClustalX alignment of *Se*Cobatoxins. Black shadows highlight residues conserved between the two isoforms. Dotted box indicates the sequences of the mature peptides [41]. Arrows indicate conserved cysteine residues from CS-αβ motif.

Differently from the other classes of CS-αβ AMPs, two different copies of cobatoxin were identified in *S*. *exigua* transcriptome (Table 1), and the corresponding mature peptides shared 41% amino acid identity. In *S*. *frugiperda*, the homolog to *Se*Cobatoxin A had been already described (acc. nr. AAP69839), whereas the homolog to *Se*Cobatoxin B was retrieved from SPODOBASE after Blast search (acc. nr. Sf1P05363-5-1). The amino acid identity between the two species ranged from 72 to 83% for cobatoxin A and B, respectively. Phylogenetic analysis including lepidopteran cobatoxin protein sequences available in GenBank revealed that at least two cobatoxins were commonly present in Noctuidae species (Fig 2) whereas no orthologs have been reported in *B*. *mori* genome so far [11].

**Fig 3:**
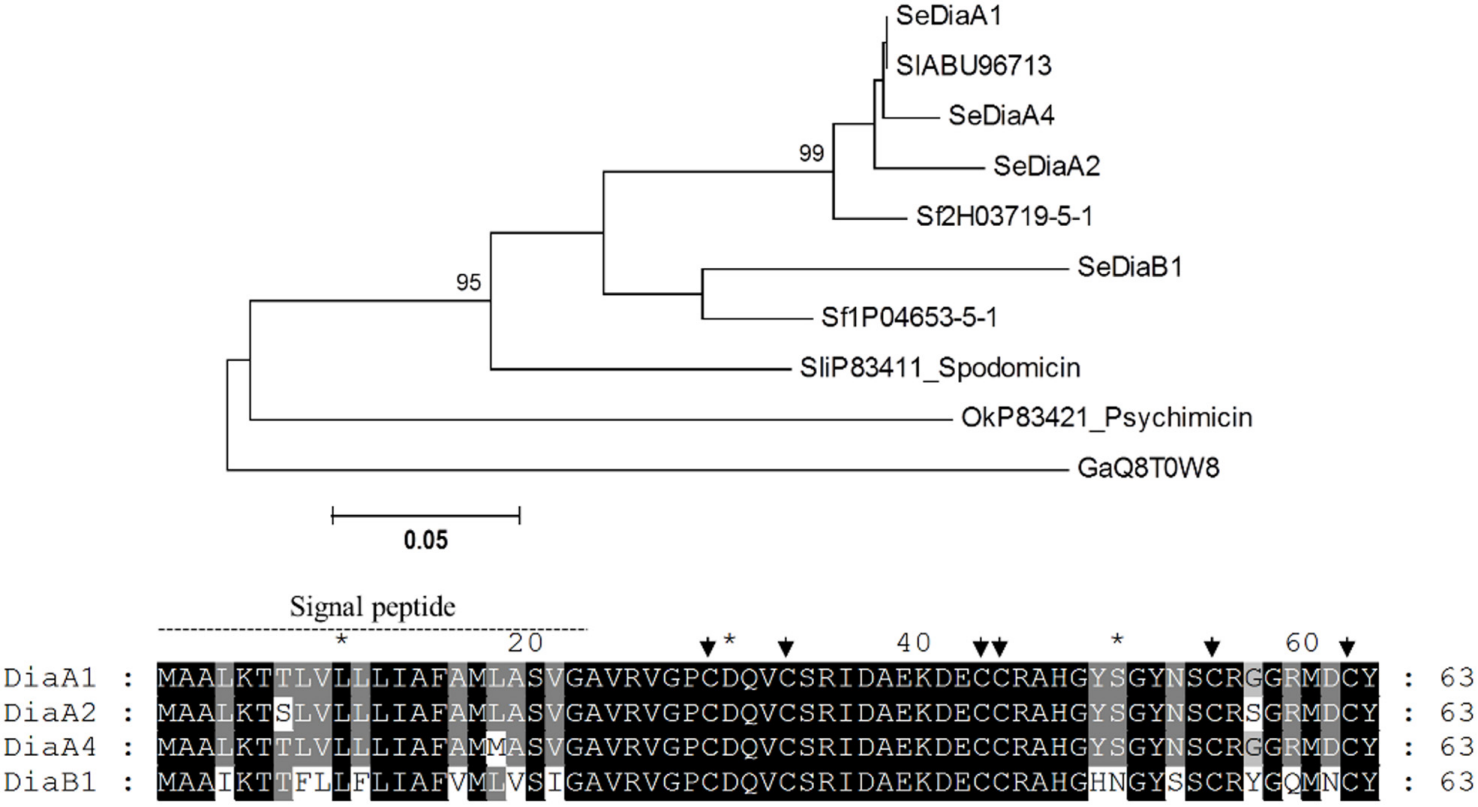
Phylogenetic analysis of diapausin proteins from insects. Upper panel) Neighbor-joining tree of insect diapausins. SeDiapausins A3, A5, A6 and A7 were omitted since they were identical to SeDiapausin A1. Bootstrap values (above 75) are indicated on each node of the tree (100 replicates). Sequences from nr database and SPODOBASE were used. Accession number of each sequence is indicated. Ga: *G*. *atrocyanea*, Ok: *Oiketicus kirbyi*, Se: *S*. *exigua*, Sf: *S*. *frugiperda*, Sli: *S*. *littoralis*, Sl: *S*. *litura*. Lower panel) ClustalX alignment of *Se*Diapausins. Black shadows highlight residues with 100% identity whereas grey shadows highlight residues with more than 80% identity. Arrows indicate conserved cysteine residues (CX_3_CX_9_CCX_10_CX_6_C motif [44]).

Other types of cysteine—rich AMPs are diapausins, which differ from CS-αβ AMPs in their primary structure, although tridimensional folding is similar [44]. The first diapausin described was identified in the leaf beetle *Gastrophysa atrocyanea* [45], and was named as diapausin because it was found highly expressed in adults during diapause. This type of AMP has been poorly described in insects; in Lepidoptera there is only one report describing a diapausin gene (from *S*. *exigua*) [46]. In the *S*. *exigua* transcriptome, we identified eight different types of transcripts coding for *Se*Diapausins (Table 1). Based on amino acid similarity, these sequences were divided into two clusters that we named *Se*Diapausin A and *Se*Diapausin B (Fig 3). Seven different transcripts were classified as diapausins type A whereas only one was assigned to the type B group. Differences among transcripts encoding *Se*Diapausins A were mainly due to variability in the 3’ UTR sequences whereas the ORFs were conserved. *Se*Diapausins A and B shared an amino acid identity ranging from 77 to 79%. The major differences between these two types of proteins were mainly due to changes in the signal peptide and in C-terminal sequences (Fig 3). Blast searches on SPODOBASE allowed us to identify two *S*. *frugiperda* transcripts coding for proteins homologous to *Se*Diapausin A (acc. nr. Sf2H03719-5-1) and B (acc. nr. Sf1P04653-5-1) (amino acid identity from 97 to 87%, respectively) (Fig 3). Interestingly, no diapausin homologs were retrieved from the *B*. *mori* genome database after Blast search.

#### Proline-rich AMPs

**Fig 4:**
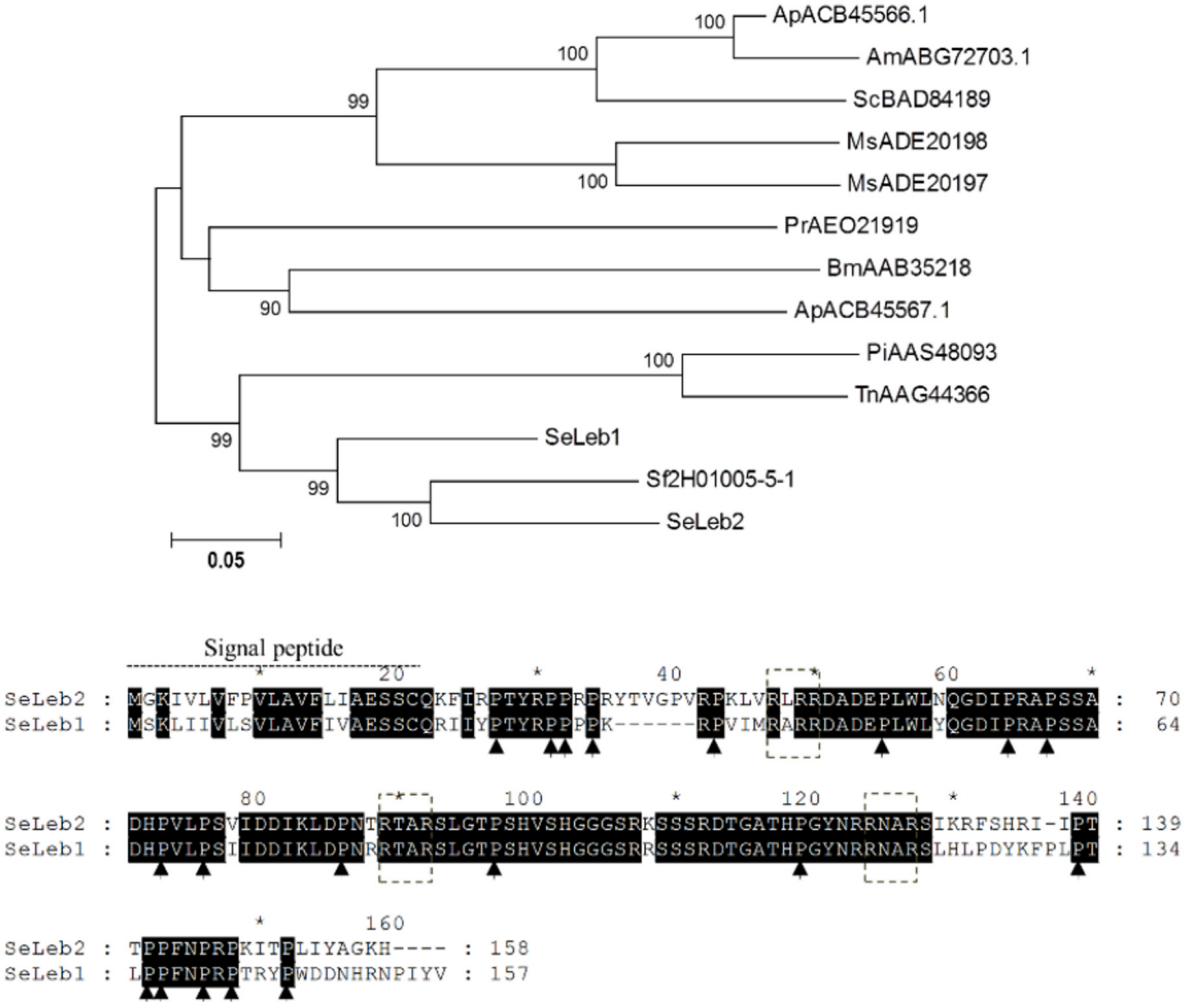
Phylogenetic analysis of lebocin proteins from Lepidoptera. Upper panel) Neighbor-joining tree of Lepidoptera diapausins. Bootstrap values (above 75) are indicated on each node of the tree (100 replicates). Sequences from nr database and SPODOBASE were used. Accession number of each sequence is indicated. Am: *A*. *mylitta*, Ap: *A*. *pernyi*, Bm: *B*. *mori*, Ms: *M*. *sexta*, Pi: *P*. *includens*, Pr: *Pieris rapae*, Sc: *S*. *cynthia*, Se: *S*. *exigua*, Sf: *S*. *frugiperda*, Tn: *T*. *ni*. Lower panel) ClustalX alignment of *Se*Lebocins. Black shadows highlight residues conserved between the two sequences, arrows indicate proline residues. Dotted boxes indicate RXXR motifs which are involved in proteolytic activation [48].

Proline-rich AMPs have a different mode of action with respect to other AMPs, since it seems that they penetrate susceptible pathogen cells and act intracellularly [47]. In Lepidoptera, the best-characterized AMPs belonging to this family are lebocins and it is commonly thought that they are synthesized as large polypeptide precursors that are subsequently split into four or five peptides by proteolytic processing [48]. Data mining of *S*. *exigua* transcriptome rendered two transcripts encoding two lebocins which shared 79% amino acid identity (Table 1). Both *S*. *exigua* lebocin amino acid sequences displayed three conserved RXXR motifs, which is important for the proteolytic activation [48] (Fig 4). Only one lebocin sequence, the *Se*Lebocin2 homolog, was found in *S*. *frugiperda* EST database (acc. nr. Sf2H01005-5-1, 80% amino acid identity). Likewise only one gene coding for a lebocin has been found in the *B*. *mori* genome [11]. Phylogenetic analysis showed that *Se*Lebocin1 is closer to *Se*Lebocin2 than to other lebocins from other lepidopteran genus (Fig 4) suggesting that these two copies may be paralogs originated from gene duplication within the S. e*xigua* species.

#### Amphipathic peptides

Amphipathic peptides are a class of α-helical AMPs present across different insect orders [2]. The first animal immune-inducible AMP described belongs to this family: it was discovered in *H*. *cecropia* and therefore was named cecropin [5]. It is thought that multiple cecropin copies are present in Lepidoptera species, and in fact, in the *B*. *mori* genome, thirteen genes have been identified so far [11]. A previous phylogenetic analysis of cecropins in *S*. *exigua* with 31 *S*. *exigua* cecropin unigenes [21] grouped them into six different subfamilies. We performed a more exhaustive analysis that included-38 *S*. *exigua* unigenes with homology to cecropins, this resulted in the identification of eight different subfamilies of transcripts, that encoded seven different pre-proteins (ORFs of *Se*Cecropin A1 and A2 were identical) (Table 1). Alignment of the deduced mature amino acid sequences encoded by the eight transcripts showed that they could be grouped into six highly similar protein classes (amino acid identities ranged from 36 to 96%) that corresponded to the six subfamilies previously described [21]. The number of protein classes increased to seven when pre-protein sequences were compared since the amino acid sequences of pre-*Se*Cecropins F1 and F2 differed by two amino acid substitutions located in the signal peptide. In the closely related species *S*. *frugiperda*, up to thirteen different types of transcripts encoding cecropins had been identified and grouped into four sub-families named A, B, C and D [49], which respectively corresponded to the families B, A, E and C described in the present study (amino acid identity ranged from 86 to 100%). However, homologs to *Se*Cecropin D (acc. nr. Sf2H07985-5-1), F1 (acc. nr. Sf1H09041-3-1) and F2 (acc. nr. Sf2H04303-5-1) are also present in *S*. *frugiperda*; their sequences were retrieved by Blast searches on SPODOBASE (amino acid identity ranged from 94 to 97%), suggesting that cecropin gene number in *Spodoptera* genus is conserved.

Another class of lepidopteran amphipathic AMPs is moricin. Moricin was first described in *B*. *mori* [50] and, differently from cecropins, it has so far only been found in Lepidoptera. *Bm*Moricin has a broad spectrum of activity since it is active against both Gram positive and negative bacteria [2]. The *B*. *mori* genome contains only one gene coding for moricin, but eight genes encoding moricin-like proteins [11]. Data mining of *S*. *exigua* transcriptome led to the identification of a unique type of transcript encoding moricin (which shared 97% amino acid identity with its *S*. *frugiperda* homolog retrieved from SPODOBASE, acc. nr. Sf2H03805-5-1) (Table 1), whereas no homologs to *B*. *mori* moricin-like proteins were found.

#### Lysozymes

Lysozymes are antimicrobial enzymes that have been extensively studied in both plant and animal kingdoms. They have the ability to cleave the β-1,4 glycosidic linkages between N-acetylmuramic acid and N-acetylglucosamine in peptidoglycans, which are components of bacterial cell walls [51]. In insects, lysozymes are divided into two categories according to the presence of catalytic amino acid residues essential for muramidase activity: the classical c-type lysozymes (c-LYZs) and the lysozyme-like proteins (LLPs) [52]. In this study 32 unigenes encoding lysozymes were clustered into seven types of transcripts that coded for 5 c-LYZs and 2 LLPs (Table 1), expanding the number of lysozyme classes previously identified in *S*. *exigua* [21]. Within the *Se*C-LYZ clusters, three types of transcripts differed in the 3’ UTR but encoded the same protein, *Se*C-LYZ1 (*Se*C-LYZ1A, 1B and 1C diverged by one amino acid substitution). The other two transcripts coded for two different proteins, *Se*C-LYZ2 and *Se*C-LYZ3. Transcripts encoding five different proteins (three c-LYZs and two LLPs) were also found in *S*. *frugiperda* [53], indicating that the lysozymes, as well as AMPs, were conserved between these two closely related species. Alignment of the predicted proteins showed that amino acid identities between *S*. *exigua* and *S*. *frugiperda* lysozymes ranged from 84 to 97% for c-LYZs and from 77 to 87% for LLPs. In the *B*. *mori* genome, one gene coding for a c-LYZ isoform and three genes coding for LLPs have been described [11].

### *S*. *exigua* midgut-specific AMPs and lysozyme transcriptional responses to *B*. *thuringiensis* toxins

**Fig 5:**
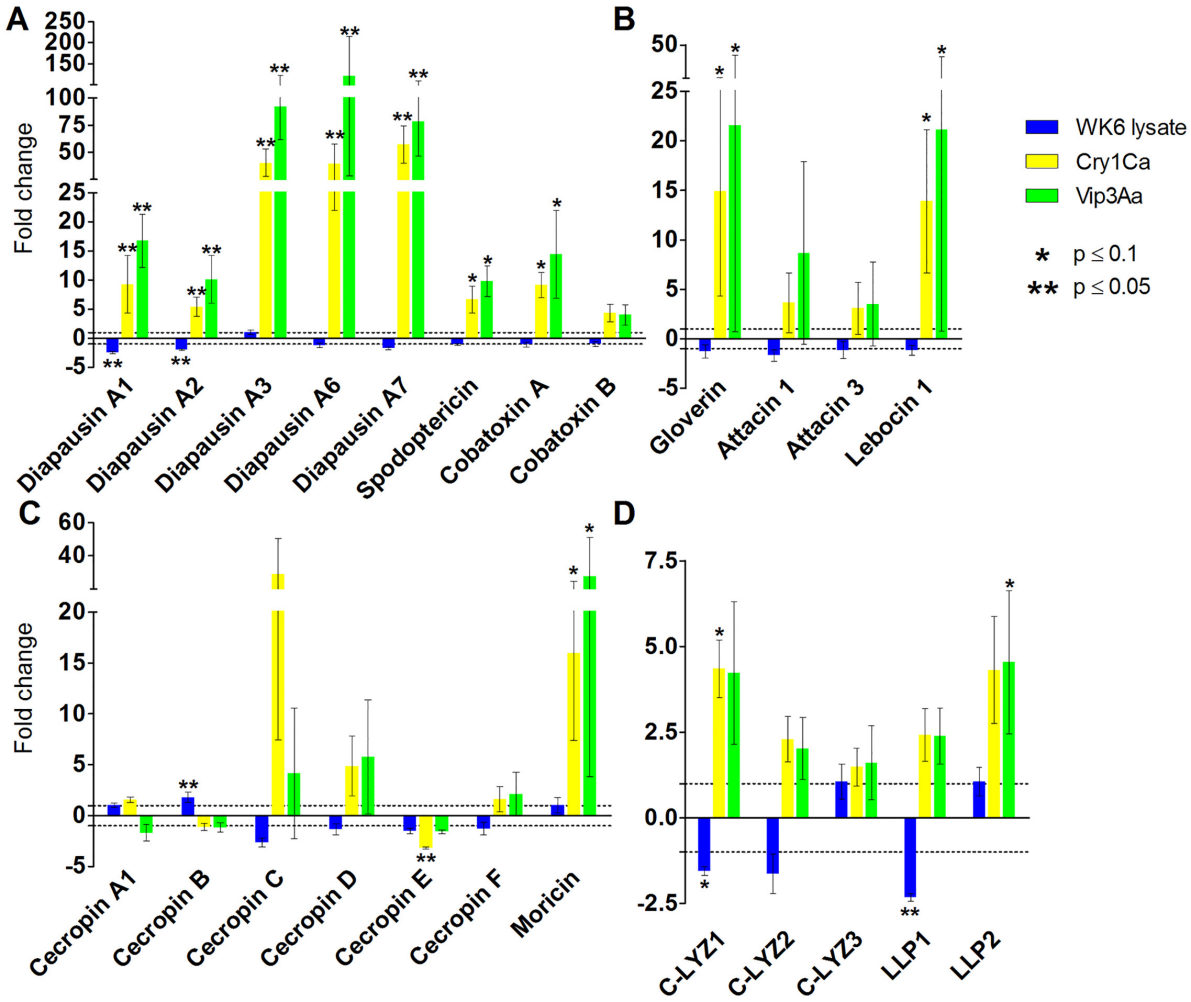
AMP and lysozyme transcript levels in *S*. *exigua* larval midgut after challenging with Bt toxins. Gene expression of each transcript after the different treatments (WK6 lysate, Cry1Ca, and Vip3Aa) was compared to its control (used as calibrator). Fold-changes were determined by using the REST MCS software. Each bar represents the mean of three independent experiments (± SEM). Dotted lines indicate 1 and -1 fold-changes. A) Cysteine-rich peptides; B) glycine- and proline-rich peptides; C) amphipathic peptides; D) lysozymes.

Gene expression changes in a large subset of AMPs and lysozymes in the *S*. *exigua* midgut were analyzed more in detail by qRT-PCR after intoxication of larvae with Bt toxins. First of all, we observed that the ingestion of *E*. *coli* WK6 proteins (present in Vip3Aa samples, and added to the Åkta-purified Cry1Ca samples) (S2 Fig) did not cause any moderate or strong changes in AMP or lysozyme gene expression (Fig 5). Although REST analysis showed that the level of four transcripts (*Se*DiapausinA1, *Se*DiapausinA2, *Se*C-LYZ1 and *Se*LLP1) significantly decreased after WK6 protein ingestion (less than 2.5-fold), and a fifth one (*Se*CecropinB) was slightly up-regulated (less than 2-fold), we considered that the overall gene regulation was not influenced by the presence of *E*. *coli* proteins in cell lysate. Moreover, WK6 proteins did not trigger any growth inhibition effects in bioassays (S4 Fig).

After ingestion of both Cry1Ca and Vip3Aa toxins at a dose inhibiting 99% of growth, almost all the transcripts coding for AMPs and lysozymes were up-regulated, showing moderate (2–20 fold) to high transcriptional activation (up than 20 fold) (Fig 5). These results are in agreement with the studies comparing transcriptional response (by transcriptome profiling or microarrays) of lepidopteran or coleopteran insects to different Cry toxins. Most of these analyses showed that, among others, genes involved in immune-response were usually up-regulated after Bt challenge, particularly genes encoding AMPs [28,32,54–61].

**Fig 6:**
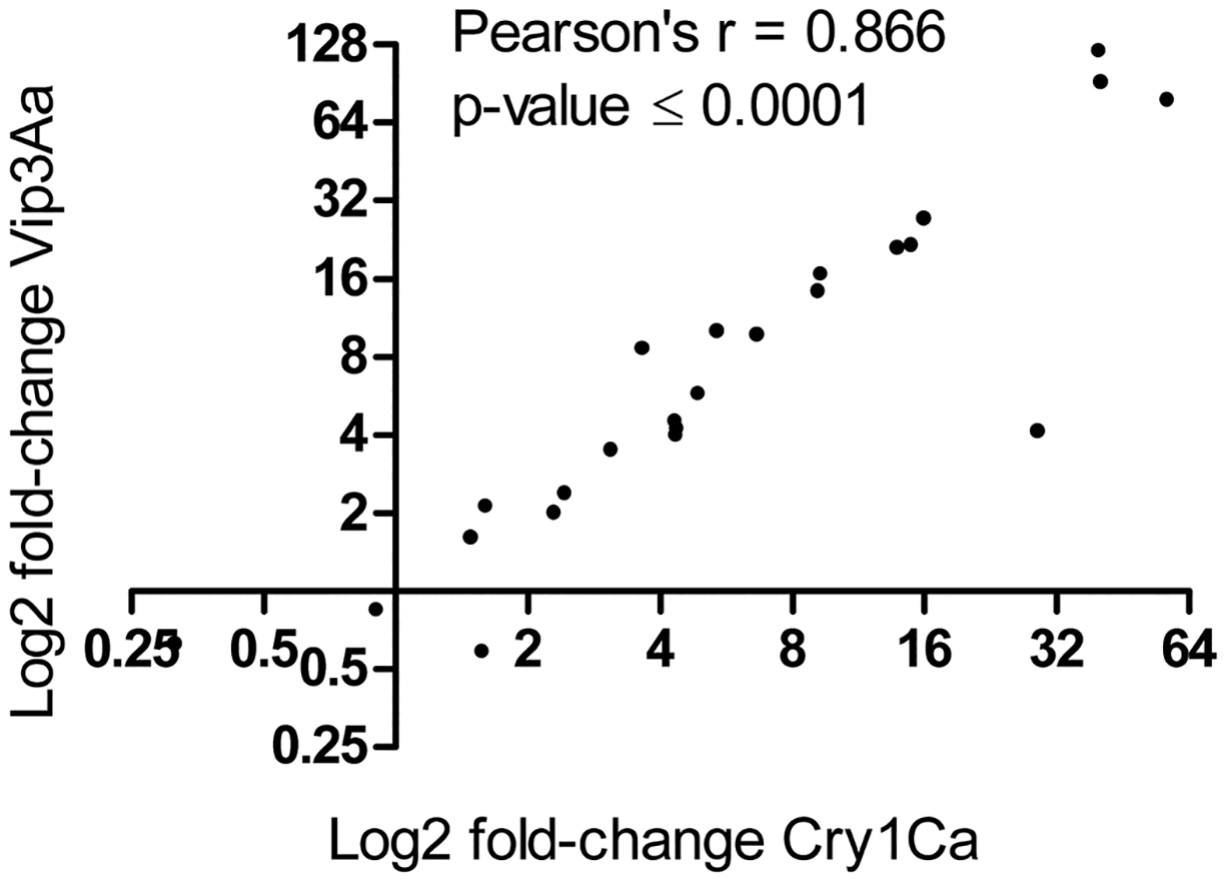
Correlation analysis of antimicrobial-protein gene expression after treatments with Cry1Ca and Vip3Aa. Correlation analysis between the average fold change (3n) after Cry1Ca and Vip3Aa treatments (Pearson r and p-value are shown).

Interestingly, AMP and lysozyme expression responded in a similar way after either Cry1Ca or Vip3Aa intoxication (Fig 6), with only few quantitative differences observed. It is worth noting that the transcriptional induction was not strictly gene family-dependent, e.g., some amphipathic peptides (*Se*Moricin) were largely up-regulated after intoxication with both toxins while others were not regulated or even down-regulated (*Se*Cecropin E) (Fig 5). Only one transcript (*Se*Cecropin E) showed a down-regulation, though slight (3-fold), after Cry1Ca ingestion (Fig 5C).

When the midgut transcriptional response to Bt toxin challenge was analyzed in detail, we observed that the most responsive AMPs were the cysteine-rich peptides. All the tested members of this family were up-regulated after ingestion of both toxins, with no major differences between Cry1Ca and Vip3Aa (Fig 5A). A more exhaustive analysis of cysteine-rich AMP response revealed that classical CS-αβ AMPs (spodoptericin and cobatoxins) showed a moderated level of induction (from 4 to 15 fold) whereas the majority of the members of diapausin family showed high levels of induction (up than 40 fold) (Fig 5A). It has been shown that classical CS-αβ AMPs are involved in humoral response against several bacteria and fungi in a number of Lepidoptera species such as *B*. *mori*, *S*. *cynthia*, *S*. *littoralis*, *S*. *frugiperda* [41,62–65]. Among these studies, only one (performed with *H*. *armigera* larvae) assessed their role in response to *B*. *thuringiensis* infection, showing that transcripts coding for defensin and a cobatoxin (homolog to *Se*Cobatoxin A) were up-regulated after intrahemocoelic injection of *B*. *thuringiensis* [66]. Interestingly, the most responsive cysteine-rich peptide family was diapausin, also one of the most expanded AMP families in *S*. *exigua* (up to 8 transcripts were retrieved in the present study). Diapausins increased their transcription 121-fold (i.e. *Se*Diapausin A6 after Vip3Aa intoxication) (Fig 5A). Similar results have been reported using microarrays to investigate transcriptional changes in *S*. *exigua* after Vip3Aa intoxication [28]. Two unigenes annotated as diapausin precursors (namely SE_U33476 and SE_U10027 that correspond to *Se*Diapausin A3 and A4, respectively) were found among the most up-regulated genes, and other two diapausins (SE_U14458 and SE_U22863, which correspond to *Se*Diapausin A2 and A1, respectively) were moderately up-regulated. Noticeably, we showed that the transcriptional response of diapausins to Cry1Ca intoxication was similar to the Vip3Aa response (Fig 6). To date, insect diapausins have received only limited study. The recombinantly-expressed *Se*Diapausin A1 showed antimicrobial activity against the Gram positive bacterium, *Bacillus megaterium* [46]. However, in the leaf beetle *G*. *atrocyanea*, transcripts encoding diapausins did not accumulate after pathogen-challenge [67]. Given the high number of transcripts coding for *Se*Diapausins that were expressed in *S*. *exigua* larval midgut and up-regulated after Bt toxin challenge and that some diapausins showed the strongest transcriptional activation, it is suggestive that these AMPs play a major role in *S*. *exigua* local response to prevent the establishment of infection and to maintain midgut immunity.

Glycine-rich and proline-rich AMPs showed inter-sample variability after Cry1Ca or Vip3Aa ingestion (Fig 5B). REST statistical analysis showed that *Se*Gloverin and *Se*Lebocin1 expression levels were significantly different from control after either Cry1Ca or Vip3Aa ingestion whereas *Se*Attacin1 and 3 did not exhibit such clear induction pattern. Interestingly, it has been shown that dsRNA-mediated silencing of *Se*Gloverin enhanced toxicity of *B*. *thuringiensis*, suggesting a role of this AMP in controlling Bt infection in *S*. *exigua* [39]. Likewise, expression of gloverin, as well as an attacin (homolog to *Se*Attacin1) and a lebocin (homolog to *Se*Lebocin1) were more frequently detected in *T*. *ni* larvae that ingested Bt preparations although, surprisingly, such transcripts were not detected in 4^th^ instar midguts [68]. In *H*. *armigera*, attacin (homolog to *Se*Attacin2/3) and gloverin increased their expression in larval body after injections of *B*. *thuringiensis* [66]. To date, glycine-rich and proline-rich peptides have been the best studied AMPs in response to Bt challenge and results indicate their involvement in response to Bt ingestion. However, their contribution to the midgut immune response (both to *B*. *thuringiensis* and to its virulence factors) needs to be clarified.

Amphipathic peptides (moricin and cecropins) showed the most divergent response to Cry1Ca and Vip3Aa (Fig 5C). Previously, up-regulation of moricin after Bt intrahemocoelic injections was observed in *H*. *armigera* larvae [66], although in *S*. *litura*, moricin was not detected in the midgut after bacterial challenge while it was induced in fat-body and hemocytes [69]. In the present study moricin showed a transcriptional response to both Cry1Ca and Vip3Aa ingestion (from 16- to 27-fold up-regulation respectively) whereas the response pattern of the other amphipathic peptides, cecropins, was not uniform. *Se*Cecropin E showed a slight down regulation only after Cry1Ca ingestion. *Se*Cecropin C and D increased their expression after both Bt toxin treatments, but the responses showed a high inter-sample variability and were not statistically significant (Fig 5C). These results led us to speculate that cecropins could play a limited role in the local immune response after Bt toxin ingestion, although they are broad-range AMPs and thought to be active against both Gram positive and negative bacteria [4,49].

Finally, four out of five lysozymes were up-regulated following both Cry1Ca and Vip3Aa treatments (Fig 5D). *Se*C-LYZ1 and *Se*LLP2 were the highest induced transcripts after both treatments (around 4.5-fold) whereas *Se*C-LYZ2 and *Se*LLP1 were only slightly induced (from 2 to 2.5-fold). *Se*C-LYZ3 was the lysozyme detected at lowest levels in all the samples tested and it never responded to Bt toxin challenge (Fig 5D). In the *S*. *exigua* close relative *S*. *frugiperda*, expression of c-LYZ and LLP homologs was tested in different tissues after intrahemocoelic injection of non-pathogenic (*E*. *coli* and *Micrococcus luteus*) and pathogenic (*Pseudomonas luminescens*) bacteria. Results showed that expression after bacterial challenge differed among the tissues tested and the only transcripts up-regulated in midguts after microbial treatments were c-LYZ1 and c-LYZ3 [53]. Likewise, in *T*. *ni*, c-LYZ expression (homolog to *Se*C-LYZ1) increased after ingestion of *B*. *thuringiensis* formulations [68] and in *H*. *armigera*, c-LYZ1 transcript level increased after injection of *B*. *thuringiensis* vegetative cells [66].

The transcriptional profile of *S*. *exigua* larval midgut after challenging with two different Bt toxins showed a wide and, in some cases, high up-regulation of transcripts coding for antimicrobial proteins, which was not substantially different between the two toxins tested (Fig 6). Both Cry1Ca and Vip3Aa are pore-forming toxins which are produced at different growth stages of *B*. *thuringiensis* as virulence factors to breach the larval midgut barrier to allow later spread into insect hemocoel [70]. More specifically, Cry toxins are important for spore toxicity whereas Vips are important for vegetative cells toxicity. Even though both toxins exert their toxicity action through the same sequence of events (activation, binding to specific receptors in the apical membrane of midgut epithelial cells and pore formation), the mode of action of these two proteins differs since they bind to different proteins [16–18], and pores seem to be structurally and functionally distinct [17]; however we observed that both trigger a common midgut immune response, which seems independent from the respective modes of action.

The cascade of events that activates the local immune response starts with the recognition of invading bacteria (like *B*. *thuringiensis*) by pathogen recognition proteins (like PGRP and GNBP), which recognize microbial molecules such bacterial peptoglycans (PGNs) or lipopolysaccharides (LPSs) [3,4]. However, in our study, we found that local immune response was not triggered after ingestion of WK6 *E*. *coli* lysate indicating any PNGs and LPS possibly present in our lysates were not influencing the immune status of *S*. *exigua* midgut. When Vip3Aa or Cry1Ca were present, a high and wide range of transcriptional activation of immune-related genes coding for AMPs or lysozymes was observed. This may be explained by the significant tissue damage that both Bt toxins provoke in susceptible larval midguts as a consequence of pore-formation and cell lysis, and by the probable production of damage-associated endogenous pattern molecules (DAMPs), which are another class of immune response elicitors [71]. Under this point of view, the local immune response observed would be triggered by the consequences of the action of the Bt proteins more than by their presence in the midgut. A recent study has shown that a large number of AMPs were activated in *Tribolium castaneum* larvae after cuticle pricking [54]. Although cuticle is a different tissue than midgut, it may be possible that the phenomenon observed is somehow correlated to what we detected in *S*. *exigua* midgut after Bt toxin injuries. Also, we should consider the presence of enteric bacteria (normally benign) that may exert pathogenic effects in case of midgut damage, and that may contribute to the activation of local immune response after pore-formation induced by the Bt toxins. An earlier study showed that loss of *M*. *sexta* gut integrity due to the action of Bt toxins enabled a gut commensal bacteria, *Enterococcus faecalis*, to translocate from the gastrointestinal tract into the hemolymph where it induced a cellular immune response and larval death [72]. Therefore, midgut production of antimicrobial proteins after wounding may be a defense mechanism used by larvae to prevent septicemia caused by a “commensal-to-pathogen” switch.

### Phenoloxidase response to *B*. *thuringiensis* Cry1Ca and Vip3Aa oral intoxication

**Fig 7:**
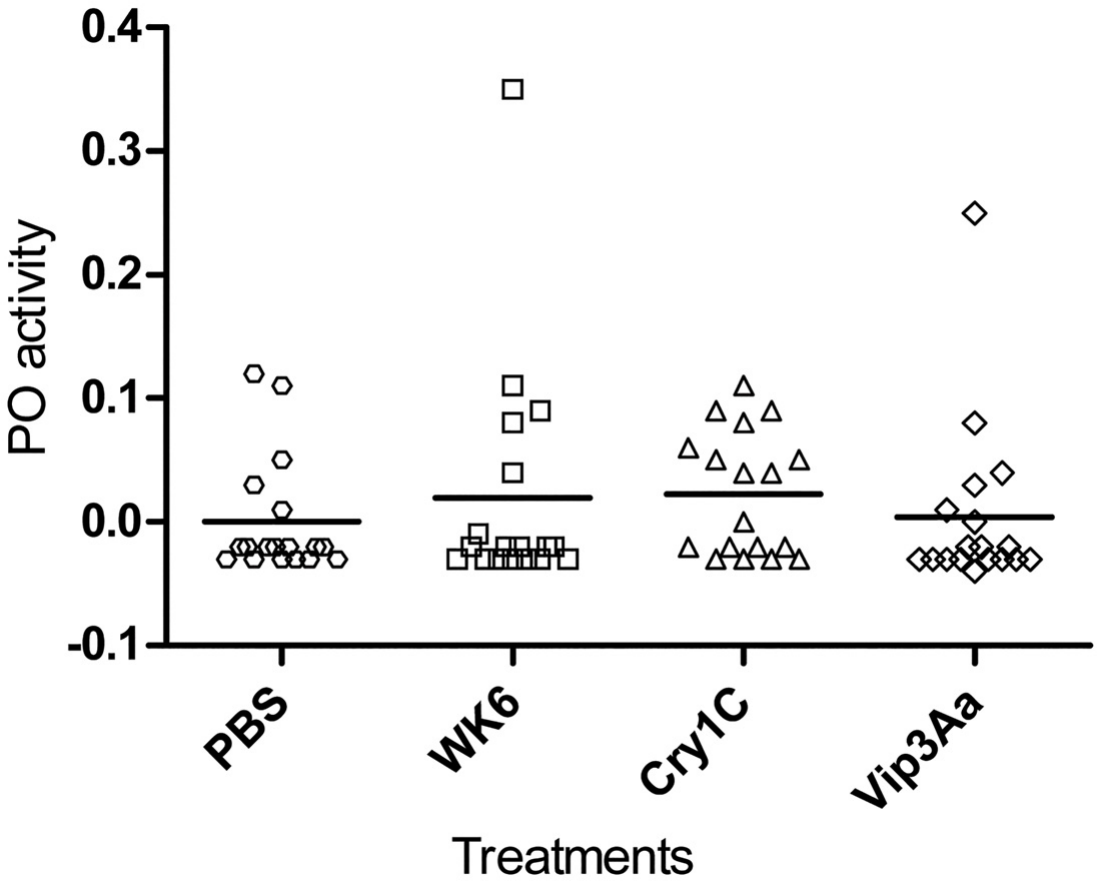
Phenoloxidase activity in the hemolymph of fourth instar *S*. *exigua* larvae after challenging with Bt toxins. Larvae were fed on artificial diet supplemented with: phosphate buffer, WK6 lysate, Vip3Aa and Cry1Ca. PO activity (absorbance increase per min per μl of hemolymph) was measured in hemolymph samples. Results represent individual values (mean of two technical replicates) and the mean of each treatment (n = 18).

In insect hemolymph, melanization is a systemic immune response controlled by a cascade of serine proteases that leads to the final activation of the pro-phenoloxidase (proPO) to its active form phenoloxidase (PO), which is responsible for the formation of melanin around intruding microorganisms [73]. The PO level is believed to be correlated with insects' immunocompetence, especially against invading fungi or insect parasitoids [74]. In the present study, the humoral response of *S*. *exigua* larvae after ingestion of a sub-lethal dose of Cry1Ca or Vip3Aa (99% growth inhibition) were investigated by measuring hemolymph PO activity. Results showed that neither the two toxins nor the WK6 lysate (used as control) had any effect on hemolymph PO activity (Fig 7), even though both Bt toxins strongly induced local (midgut) up-regulation of transcripts encoding immune response effectors.

The absence of PO response (measured 24 h after Bt toxin ingestion) may be due to the lack of bacterial infection in the hemolymph at that time, since no Bt vegetative cells or spores able to colonize insect body were present in our preparation. Freitak *et al*. [74] showed that in *T*. *ni*, after ingestion of a non-pathogenic bacterial cocktail, expression of seventeen-immune related genes (among which ten AMP and lysozyme transcripts) were up-regulated in the midgut whereas PO activity in hemolymph was even lower than in control larvae. This may suggest a local response activated in Lepidoptera larval midgut when a higher bacterial load or wounding are detected, but does not imply an automatic activation of the systemic response. Such a response may be later triggered only when bacteria reaches the body cavity as a consequence of a successful infection establishment.

In Lepidoptera, few studies have recorded PO activation after Bt oral ingestion and they are somewhat contradictory. For example, in *G*. *mellonella* both LC_15_ and LC_50_ doses of Bt spores and crystals resulted in significantly elevated hemolymph PO levels compared to control (even if PO activity was lower after exposure to LC_50_ than to LC_15_ dose) [75]. Other authors reported that ingestion of a mix of spores and crystals significantly increased hemolymph PO activity in *Plodia interpunctella* whereas in *H*. *virescens* and *T*. *ni* the increase was around 2-fold. Notably, no increase was observed for *S*. *exigua* but it was the only species that showed a 20% mortality at the used Bt dose, compared with 50% mortality of the other insects [76]. In another study with *T*. *ni*, hemolymph PO activity in susceptible larvae was even reduced after ingestion of a sub-lethal doses of a mix of spores and crystals [77]. Some authors speculated that the reduction in PO activity after ingestion of sub-lethal doses of Bt spores and crystals could be due to the contamination of the hemolymph by bacterial cells which can overwhelm the cellular immune-reactions by depleting the hemocytes (which are the main responsible for proPO production) [75].

### *S*. *exigua* midgut-specific AMP and lysozyme transcriptional responses to baculovirus challenge

**Fig 8:**
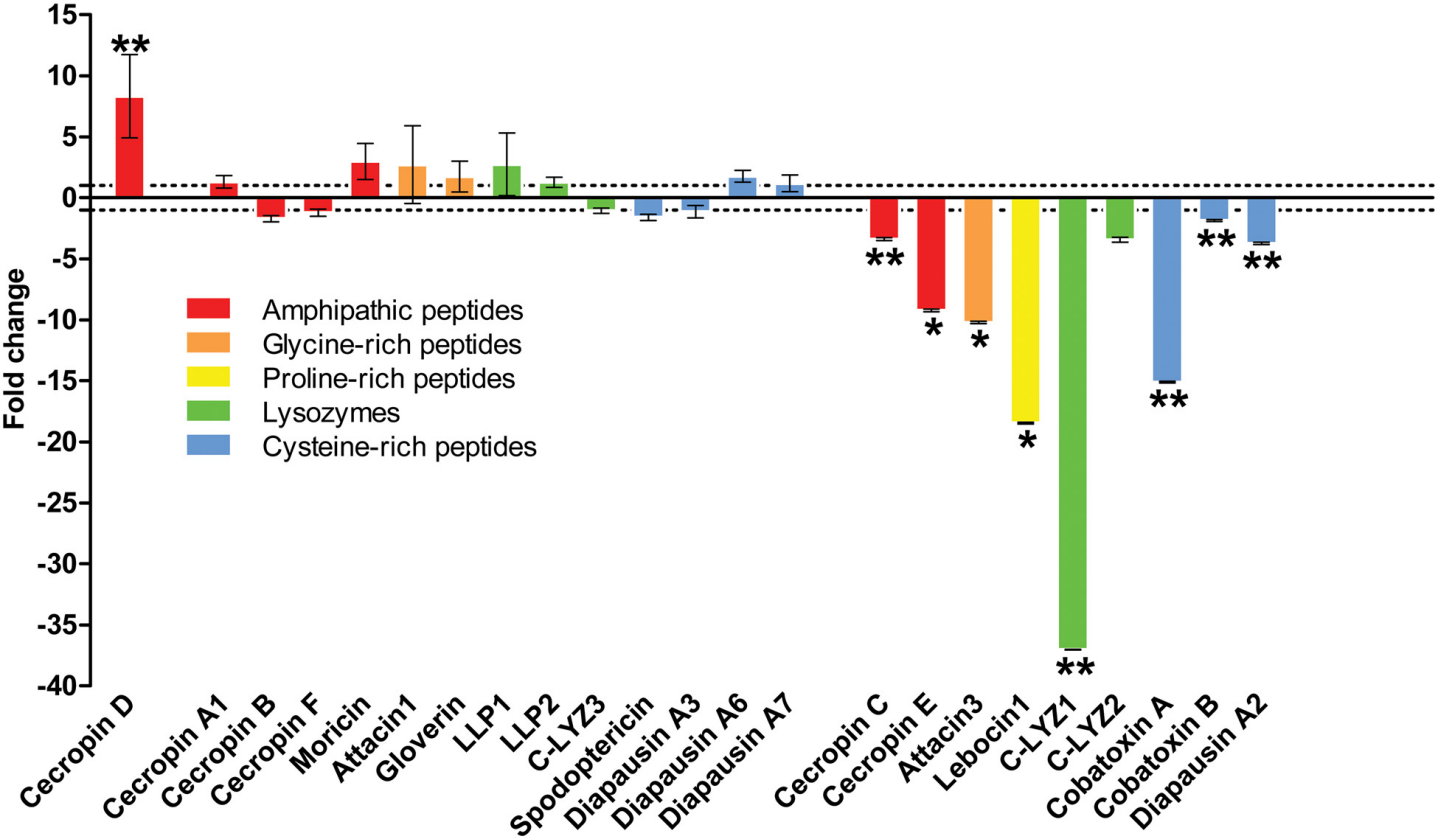
AMP and lysozyme transcript levels in *S*. *exigua* larval midgut after challenging with SeMNPV. Gene expression of each transcript after baculovirus treatment was compared to its control (used as calibrator). Fold-changes were determined by using the REST MCS software. Each bar represents the mean of three independent experiments (± SEM). Dotted lines indicate 1 and -1 fold-change. * p-value < 0.1; ** p-value < 0.05.

The transcriptional response of AMPs and lysozymes was also analyzed in *S*. *exigua* midgut after baculovirus infection. Results showed that only one out of 23 transcripts coding for effector molecules was up-regulated after viral infection, namely *Se*Cecropin D, whose transcript was 8-fold more abundant than in control larvae (Fig 8). Expression of the remaining effectors was down-regulated or did not show a response to the infection. This response was in agreement with that described by Jakubowska *et al*. [31] working with guts of *S*. *exigua* baculovirus infected larvae, where a down- or a non-regulation of immune-related gene expression (including four genes encoding AMPs) was observed. In the present study, the highest levels of repression were observed for *Se*C-LYZ1 (37-fold reduction), followed by *Se*Attacin3 (18-fold), *Se*Cobatoxin A (15-fold), and *Se*Attacin3 and *Se*CecropinE (around 10-fold). *Se*DiapausinA2, *Se*CecropinC and *Se*C-LYZ2 were the least down regulated transcripts (around 4-fold reduction). It is noteworthy that decreases in transcription after viral infection were not family-dependent, since in each antimicrobial protein family, only few members were specifically down-regulated. The overall picture that emerged from this comprehensive analysis of immune effector expression regulation after baculovirus infection was that only few peptides or enzymes with antimicrobial activity were transcribed in midguts of infected larvae (with the exception of *Se*Cecropin D) in contrast to what happened after Bt toxin ingestion. Manipulation of the host local immune response has been proposed as a method that would improve baculovirus fitness by promoting the increase of enteric bacteria load in the midgut [31]. Our results are in agreement with this hypothesis, since after baculovirus infection, a microbial-friendly environment is maintained in *S*. *exigua* midgut.

## Conclusions

This work has addressed the response of local (midgut) immune system effectors in *S*. *exigua* after ingestion of different virulence factors (Bt toxins such Cry1Ca and Vip3Aa) and a viral pathogen (SeMNPV) used in microbial control agricultural practices. Here, we have shown that larvae responded locally to the toxic effects associated to the Bt protein by widely inducing transcription of their AMP and lysozyme arsenal in the midgut most probably to prevent septicemia produced by commensal bacteria, without the contemporaneous activation of the systemic defense (represented by PO activity). On the other hand, baculovirus infection led to a down-regulation of some AMPs and lysozymes in the midgut and to no or little changes in the other ones, confirming the importance for the virus to establish its infection in a “microbial-friendly” gut environment to increase its fitness [31]. Taken together, these results show the importance of the midgut immune response in shaping the first step of infections triggered by ingested pathogens, thus highlighting the need to take it into account in host-pathogen interaction studies.

## Supporting Information

S1 Fig Growth inhibition dose-response curve of *S*. *exigua* newly molted L4 larvae challenged with Cry1Ca. Growth inhibition values were calculated following Herrero *et al*. [30]. Three biological replicates of the experiment (using 8 larvae per dose) were performed.
(TIF)

S2 Fig SDS-PAGE profiles of Bt proteins and controls used for *S*. *exigua* L4 treatments. Lane 1) crude WK6 lysate containing 1 μg Vip3Aa; 2) crude empty WK6 lysate; 3) 1 μg of Åkta purified Cry1Ca; 4) 1 μg of Åkta purified Cry1Ca mixed with crude empty WK6 lysate; 5) Precision Plus Protein Unstained Standards (Bio-Rad, hercules, CA, US).
(TIF)

S3 Fig ClustalX alignment of Lepidoptera x-tox proteins. Black box indicates the signal peptide sequences; dotted black boxes indicate CS-αβ motifs. In each motif, conserved six cysteines are highlighted in green. Accession number of each sequence is indicated. Bm: *B*. *mori*, Ha: *H*. *armigera*, Hv: *H*. *virescens*, Se: *S*. *exigua*, Sf: *S*. *frugiperda*.
(TIF)

S4 Fig Comparison of growth rate values from larvae that ingested phosphate buffer or WK6 lysate proteins. Each dot represents an individual observation. Lines represent mean ± SD. Mann-Whitney U and p-value are shown.
(TIF)

S1 Table Blastn results using S. exigua antimicrobial proteins as query and S. exigua Sanger library as database. S. exigua Sanger sequences acc. nr. from JK786560 to JK789862. (XLSX)

S2 Table Primers for sequence confirmation, qRT-PCR analyses and/or transcript detection. (XLSX)

S3 Table Efficiencies of primers used for qRT-PCR analyses. (XLSX)
