# Supplementary figures and images for: Dissimilar Regulation of Antimicrobial Proteins in the Midgut of Spodoptera exigua Larvae Challenged with Bacillus thuringiensis Toxins or Baculovirus
Source: PLoS One. 2015 May 18;10(5):e0125991. doi: 10.1371/journal.pone.0125991 (PMC4436361; doi:10.1371/journal.pone.0125991)

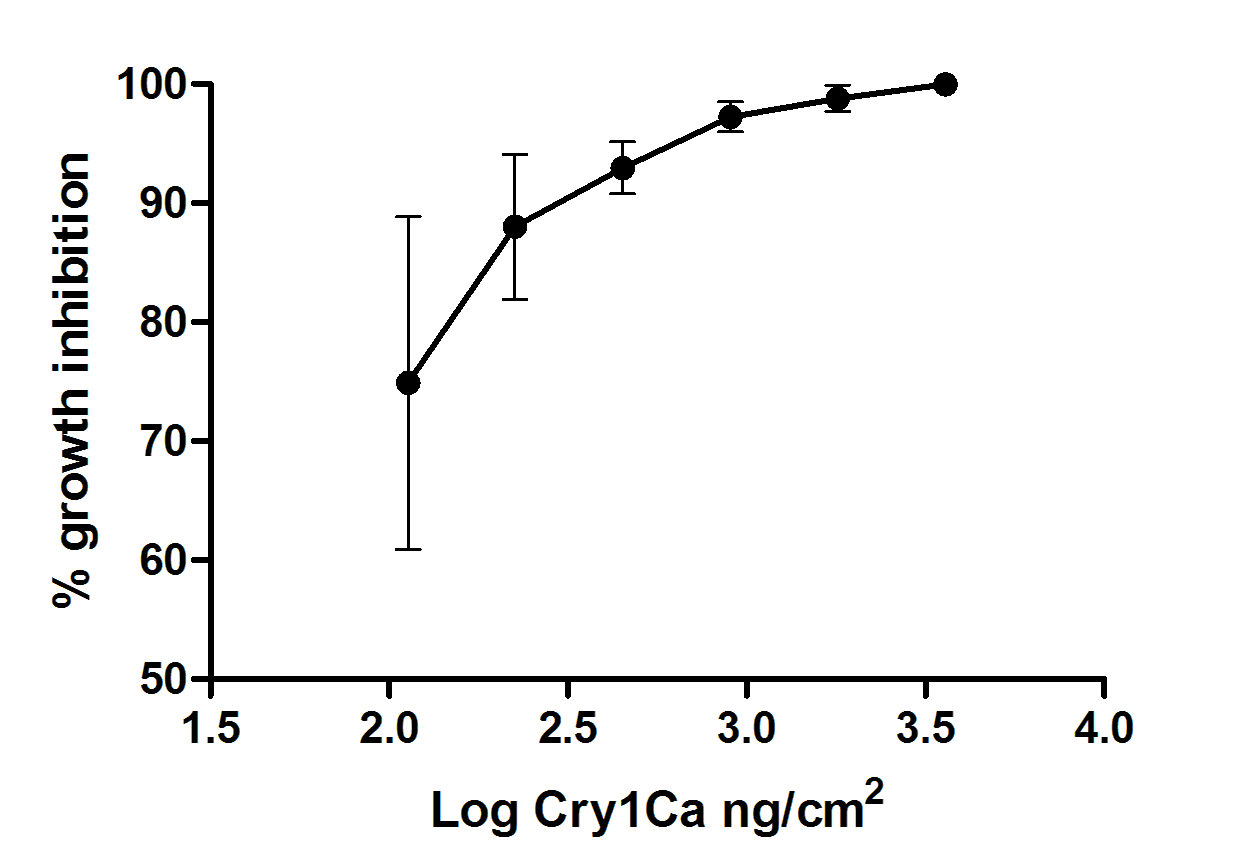

Supplement: S1 Fig — Growth inhibition values were calculated following Herrero et al. [30]. Three biological replicates of the experiment (using 8 larvae per dose) were performed. (TIF) [file pone.0125991.s001.tif]

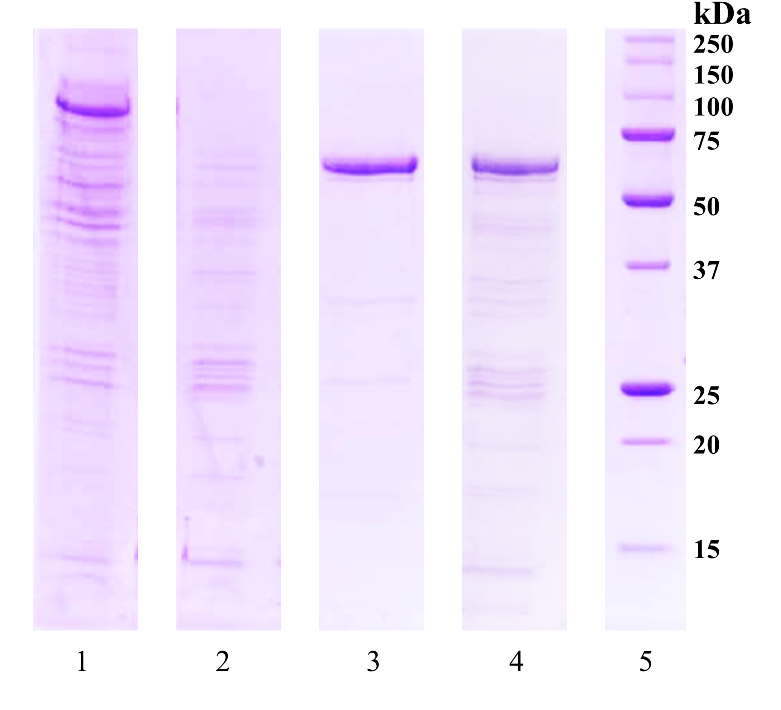

Supplement: S2 Fig — Lane 1) crude WK6 lysate containing 1 μg Vip3Aa; 2) crude empty WK6 lysate; 3) 1 μg of Åkta purified Cry1Ca; 4) 1 μg of Åkta purified Cry1Ca mixed with crude empty WK6 lysate; 5) Precision Plus Protein Unstained Standards (Bio-Rad, hercules, CA, US). (TIF) [file pone.0125991.s002.tif]

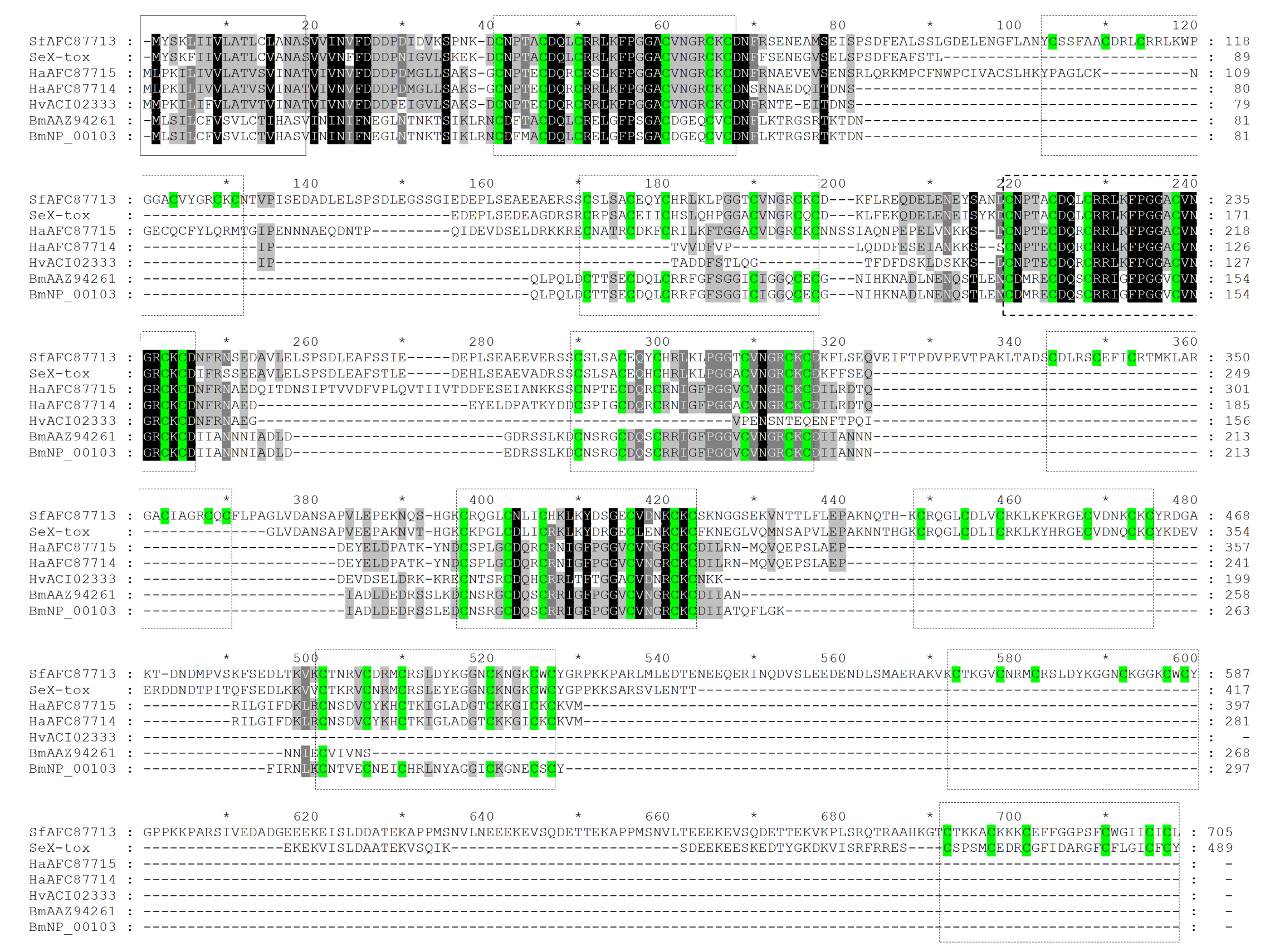

Supplement: S3 Fig — Black box indicates the signal peptide sequences; dotted black boxes indicate CS-αβ motifs. In each motif, conserved six cysteines are highlighted in green. Accession number of each sequence is indicated. Bm: B. mori, Ha: H. armigera, Hv: H. virescens, Se: S. exigua, Sf: S. frugiperda. (TIF) [file pone.0125991.s003.tif]
